# Intratumoral Heterogeneity Determines the Expression of mTOR-pathway Proteins in Prostate Cancer

**DOI:** 10.1155/2019/1296865

**Published:** 2019-12-11

**Authors:** Giorgio Ivan Russo, Jörg Hennenlotter, Ulrich Vogel, Ursula Kühs, Thomas Manfred Wurm, Valentina Gerber, Tim Neumann, Sebastiano Cimino, Arnulf Stenzl, Tilman Todenhöfer

**Affiliations:** ^1^Urology Section, Department of Surgery, University of Catania, Catania, Italy; ^2^Department of Urology, Eberhard Karls University of Tübingen, Tübingen, Germany; ^3^Department of Pathology, Eberhard Karls University of Tübingen, Tübingen, Germany; ^4^Studienpraxis Urologie, Nuertingen, Germany

## Abstract

The aim of this study was to evaluate the expression of mammalian target of rapamycin (mTOR), phosphorylated-mTOR (p-mTOR), and eukaryotic translation initiation factor 4E-binding protein 1 (4E-BP1) in prostate cancer (PCa) in order to assess intratumoral heterogeneity and correlation with clinicopathological parameters. Tissue samples from 115 patients undergoing radical prostatectomy were included in a tissue microarray comprising (A) tissue from the tumor center, (B) malignant border of the tumor, (C) tumor-adjacent benign tissue, and (D) tumor-distant benign prostatic tissue. Immune reactive scores 0-12 were correlated with clinical data in reference to localization. A meta-analysis of studies investigating the association between biochemical recurrence (BCR) and parameters of the mTOR pathway was conducted. Regardless of the location within the tumor, cancer tissue showed higher expression of mTOR, p-mTOR, and 4EB-P1 compared to benign tissue (*p* < 0.01). Significant differences in expression between tissue samples from groups C and D were observed for mTOR and p-mTOR. When considering expression according to the pathological stage, we observed lower p-mTOR expression in pT3 vs. pT2 (7.9 and 6.3; *p* = 0.01). After a median follow-up of 74.5 months (IQR 65.0-84.0), 27 patients (23.47%) developed BCR. Weak staining of mTOR was associated with shorter time to BCR (HR: 2.0; *p* = 0.049) after correcting for PSA and T stage. However, a significant association of mTOR expression with BCR was found for specimens from the malignant border of the tumor (B) but not the tumor center (A) (*p* = 0.0034 log rank). In a meta-analysis, we found that the expressions of mTOR ((RR) = 0.70; 95% CI 0.43-1.12; *p* = 0.13) and 4E-BP1 ((RR) = 0.86; *p* = 0.53) were not statistically associated with BCR, while strong staining of p-mTOR was associated with a lower risk of BCR ((RR) = 0.57; *p* = 0.002). All 3 markers showed stronger expression in PCa and exhibited local gradients in relation to the border of tumor and healthy tissue. Our results suggest an important role of intratumor heterogeneity for the use of mTOR parameters as biomarkers in PCa.

## 1. Introduction

Prostate cancer (PCa) represents the most common cancer in men in developed countries in 2013 [1]. In recent decades, the goal of precision cancer medicine has been to pair clinical and biologic data to provide better and more efficient treatment options for cancer care [2]. Tissue microarrays have been established as an important tool for biomarker analysis. In fact, TMA is useful to discover molecular aberrations in different regions of a tumor, defined as intratumor heterogeneity (ITH), having critical implications in precise diagnosis and the treatment of cancers [3].

The phosphatidylinositol 3-kinase/proteinkinase B/mammalian target of rapamycin pathway (PI3K/Akt/mTOR pathway) has long been known to play an important role in the development of PCa [4]. The mTORC1 complex signals primarily through effectors, including phosphorylation of the 4E-binding protein (4E-BP1), leading to an increase in cap-dependent translation and promoting proliferation [5].

In response to extracellular stimuli, mTOR is activated by the phosphorylation of Ser2448 through the PI3K/Akt/mTOR pathway [6, 7]. The dysregulation of mTOR plays a crucial role in tumorigenesis, angiogenesis, cellular growth, and metastasis [8]. For these reasons, the PI3K/Akt/mTOR pathway has emerged as a potential candidate serving as a therapeutic target for treatment of solid tumors.

Tumor heterogeneity has an important impact on the potential implications of biomarkers. To date, only few data exists on the impact of tumor heterogeneity on the potential prognostic role of mTOR parameters as biomarkers in PC [9, 10]. Moreover, the prognostic role of these biomarkers in the context of biochemical recurrence after radical prostatectomy is not fully understood.

The aim of the present study was to evaluate intratumoral heterogeneity of the expression of mTOR, phosphorylated-mTOR (p-mTOR), and eukaryotic translation initiation factor 4E-binding protein 1 (4E-BP1) in patients with PCa using the TMA technique. We also aimed to compare our results with the public PC RNA-seq data set from The Cancer Genome Atlas (TCGA) and to estimate the prognostic role of these biomarkers in a meta analysis.

## 2. Material and Methods

### 2.1. Patients' Samples

Tissue samples from 115 consecutive patients who underwent radical prostatectomy were constructed for a TMA using 1 core per localization. Clinical data including age, preoperative PSA, Gleason score, pathological stage, lymph node status, and metastatic disease were included. Patients who underwent neoadjuvant hormonal therapy were excluded from the study. Patients were staged and graded according to TNM staging on prostate cancer. The study received IRB approval (290/2010BO2), and it has been conducted in accordance with the Declaration of Helsinki (1964).

### 2.2. Tissue Microarray and Immunohistochemistry

**Figure 1:**
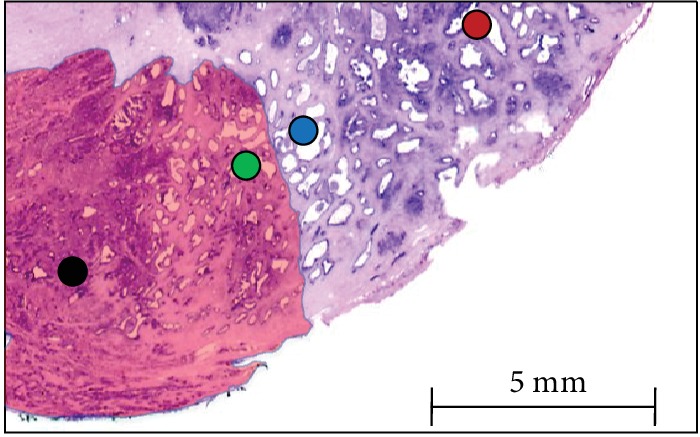
Sites of the samples in relation to the tumor area (red hatches) with the corresponding dots displayed on the TMA carrier. Tumor zone (sample A, black dot), malignant tissue of the tumor invasion front (sample B, green dot), benign tissue adjacent to the tumor invasion front (sample C, blue dot), and benign tissue (sample D, red dot). Hematoxylin-eosin-stained slide.

Specimens were HE stained, and specific areas were selected for inclusion in the TMA. In each patient, four localizations were included in the TMA: (A) tissue from the tumor center, (B) the malignant border of the tumor, (C) benign tissue adjacent to the tumor, and (D) tumor-distant benign prostatic tissue (Figure 1). The process was performed as previously reported [11, 12].

Immunohistochemistry on consecutive sections of the TMA was performed in order to investigate the expression of mTOR, phosphorylated-mTOR (p-mTOR), and eukaryotic translation initiation factor 4E-binding protein 1 (4E-BP1).

Staining was performed according to the following protocol: Sections were cut at 5 *μ*m, transferred to slides (SuperFrost Plus, Langenbrinck, Teningen), deparaffinized in xylene, and rehydrated in an ascending alcohol series. Antigen retrieval was performed by boiling in 10 mM citrate buffer in a microwave oven. To eliminate endogenous alkaline phosphatase, a dual endogenous block (Dako, Glostrup, Sweden) was used. Slides were incubated with rabbit monoclonal antibodies against mTOR (Cell Signaling Technology, Beverly, MA, USA), p-mTOR (Ser2448, Cell Signaling), and 4E-BP1 (Cell Signaling) at a dilution of 1 : 25, 1 : 50, and 1 : 100, respectively, in an antibody diluent for 1 h at room temperature.

The EnVision™ G/2 System/AP test kit (Dako) and Chromogene Permanent Red were used for visualization. The slides were counterstained with hematoxylin and mounted with Aquatex (Merck, Germany). For a negative control, a primary antibody was omitted.

**Figure 2:**
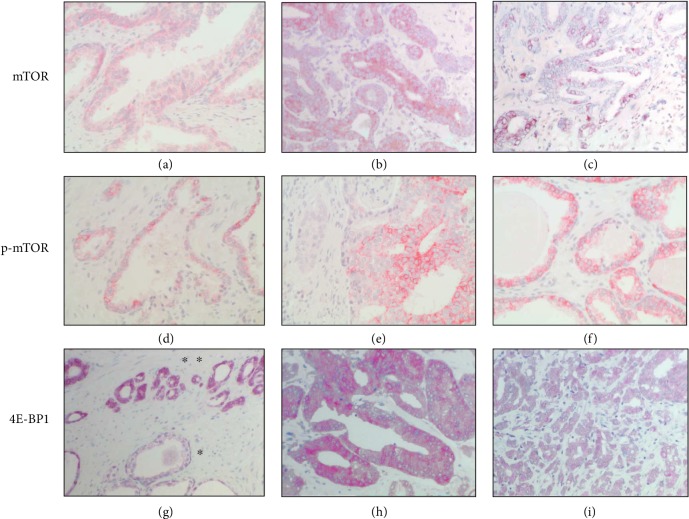
(a–i) Representative mTOR, p-mTOR, and 4E-BP1 staining results: (a, d) normal prostate tissue; (g) normal tissue (∗) adjacent to prostate cancer tissue (∗∗); (b, e, h) prostate cancer tissue with strong expression; (c, f, i) prostate cancer tissue with weak expression. 40x magnification.

All TMA slides were evaluated in a blinded manner by two investigators (T.M.W. and V.G.), and intensity as well as frequency of staining were semiquantitatively classified according to an immune reactive score of 1-12 [13] (see Figure 2).

### 2.3. Follow-Up Assessment

Following radical prostatectomy, follow-up of each patient, including PSA measurements and clinical monitoring according to the EAU guidelines, were assessed using patients' files. Two consecutive values of PSA > 0.2 ng/ml were defined as biochemical recurrence (BCR). Metastatic disease was defined as the occurrence of metastasis during the follow-up through standard imaging and cancer-specific mortality as death due to PCa.

### 2.4. Analysis of mTOR and 4E-BP1 from TCGA

To evaluate representative RNA expression patterns of mTOR and 4E-BP1, we analyzed the public PC RNA-seq data set from The Cancer Genome Atlas (TCGA) [14] and compared RNA and protein expression of mTOR and 4E-BP1 according to the T stage and Gleason score. Protein expression levels in TCGA were derived through a reverse phase protein array (RPPA). Proteins are extracted from tumor tissue or cultured cells, denatured by SDS, and printed on nitrocellulose-coated slides followed by an antibody probe [14].

### 2.5. Meta-Analysis of the Literature

This analysis was conducted according to the Preferred Reporting Items for Systematic Reviews and Meta-Analyses (PRISMA) guidelines [15]. We performed a systematic literature search in the PubMed, Embase, Cochrane, and Academic One File databases using Medical Subject Headings (MeSH) indexes, keyword searches, and publication types until August 2017. The search was limited to English language articles. The search terms included “prostate”, “prostate cancer”, “prostate specific antigen”, “mTOR”, “phosphorylated mTOR”, “4-Eukaryotic binding protein 1”, and “biochemical recurrence”. Reference lists in relevant articles and reviews were also screened for additional studies.

### 2.6. Statistical Analysis

Continuous variables are presented as the mean ± standard deviations, and differences between groups were tested by Student's independent *t*-test or the Mann-Whitney *U* test on the basis of their normal or nonnormal distribution, respectively (the normality of the variables' distribution was tested by the Kolmogorov-Smirnov test). Multiple comparisons were performed by the Kruskal-Wallis test or ANOVA with the post hoc Bonferroni test.

Protein expression of mTOR, p-mTOR and 4E-BP1 and the mRNA expression of the TCGA cohort were stratified according to their respective median into “weak” (<median) and “strong” (≥median). Kaplan-Meier analysis and log-rank tests were performed to calculate associations with BCR.

Cox regression analysis was performed to identify predictive factors of BCR, metastatic disease, and cancer-specific mortality after adjusting for age, PSA, pathological stage, and Gleason score. All tests were completed using SPSS software, version 19 (SPSS Inc., IBM Corp, Somers, NY). For all statistical comparisons, significance was considered *p* < 0.05.

For the meta-analysis, the associations between immunostaining and BCR were evaluated by calculating the Ln (relative risk). Statistical heterogeneity was assessed using the Cochran *Q* and *I*^2^ statistics. The analysis was performed using RevMan software v.5.1 (Cochrane Collaboration, Oxford, UK).

## 3. Results

The median patient age was 65.0 years (interquartile range (IQR): 60.8-70.0), and the median PSA value was 7.8 (IQR: 4.9-12.9). In total, pathological stage pT3 was found in 37 patients (32.2%), while 53 patients (46.1%) had a high Gleason score (GS) from 7 to 10 (see Table 1). The median follow-up time was 74.5 months (IQR 65.0-84.0 months).

All 115 samples were stained successfully for mTOR, p-mTOR, and 4E-BP1 allowing adequate microscopic evaluation. Expression was exclusively located in the cytoplasm for all three markers, where no nuclear staining was observed. Representative staining results are given in Figure 2.

Median staining scores of mTOR, p-mTOR, and 4E-BP1 were 9.5 (IQR: 8.0-12.0), 8.0 (IQR: 4.0-10.0), and 8.0 (IQR: 6.0-11.2), respectively.

**Figure 3:**
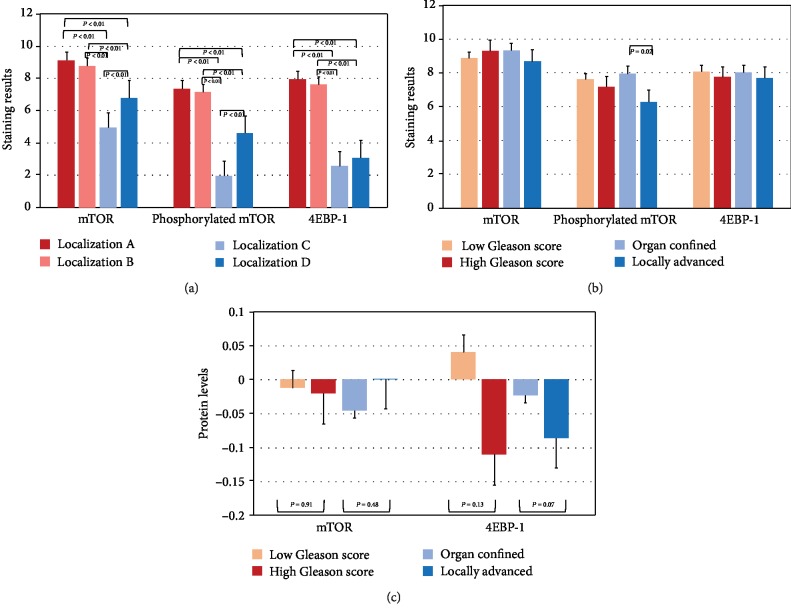
(a) Mean staining results of mTOR, phosphorylated mTOR, and 4E-BP1 in (A) tissue samples located in the tumor center, (B) in the tumor invasion front, (C) benign tissue samples adjacent to the tumor invasion front, and (D) tumor-distant remote tissue. Post hoc analyses for each comparison were performed with the Bonferroni test. (b) Mean staining results of (tumor tissue samples A, see text) for mTOR, phosphorylated mTOR, and 4E-BP1 according to Gleason score and pathological stage in our own cohort. (c) Protein levels according to the reverse phase protein array of mTOR and 4E-BP1 according to Gleason score and pathological stage in the TGCA cohort.

Expressions of mTOR, in samples of location A, B, C, and D, revealed mean expression scores of 9.1 (SD: 2.9), 8.8 (SD: 3.3), 5.0 (SD: 3.8), and 6.8 (SD: 3.7), respectively (between groups: *p* < 0.01). p-mTOR expression was 7.4 (SD: 3.5), 7.2 (SD: 3.6), 2.0 (SD: 2.6), and 4.6 (SD: 3.5) (between groups: *p* < 0.01), respectively, while 4E-BP1 expression was 8.0 (SD: 3.3), 7.6 (SD: 3.2), 2.6 (SD: 2.0), and 3.1 (SD: 2.6) (between groups: *p* < 0.01), respectively (Table 2). In the multiple comparison between groups, we found significant differences in the expression of the markers in the respective location groups (A-D) (*p* < 0.01). Expression of the markers in samples from locations A and B showed no significant differences. Moreover, expression of 4E-BP1 did not differ significantly between locations C and D (Figure 3(a)). We did not find significant differences between high and low Gleason scores. However, when considering expression according to pathological stage, we observed lower p-mTOR expression in pT3 vs. pT2 (7.9 and 6.3; *p* = 0.01, Figure 3(b)), whereas no differences were observed for mTOR (9.3 and 8.7; *p* = 0.67) and 4E-BP1 (8.0 and. 7.7; *p* = 0.47).

### 3.1. In Silico Analysis of mTOR and 4E-BP1 Expression from TCGA

A potential association of mTOR and 4E-BP1 mRNA and protein levels with the pathological stage and Gleason score has also been investigated using The Cancer Genome Atlas data [14]. mRNA expression of both mTOR (723.8 vs. 1100.3; *p* = 0.02) and 4E-BP1 (2596.7 vs. 2030.4; *p* = 0.03) was significantly lower in tumors with high Gleason scores.

**Figure 4:**
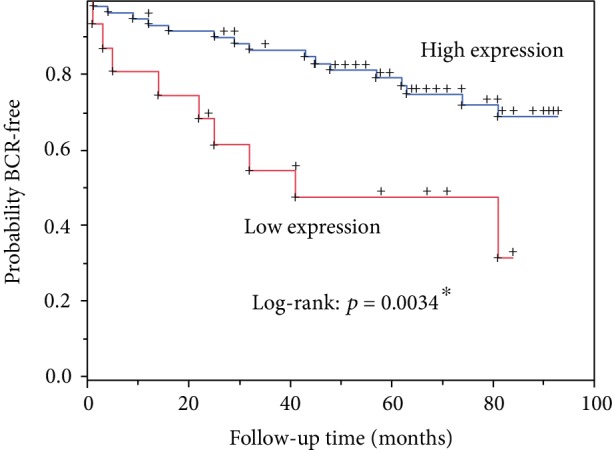
Kaplan-Meier curves for mTOR expression in localization B dichotomized by the median into low and high expression.

Using linear regression analysis, protein levels and mRNA expressions were positively associated both for mTOR (*r* = 0.365; *p* < 0.01) and 4E-BP1 (*r* = 0.234; *p* < 0.01) (Figure 4).

### 3.2. Role of m-TOR, p-mTOR, and 4E-BP1 as Prognostic Factors of BCR

**Figure 5:**
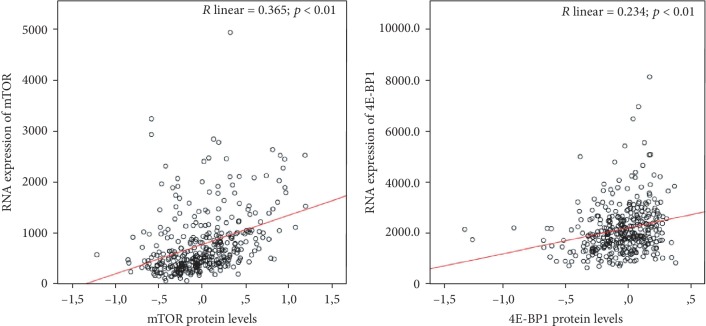
Regression analysis between protein levels and RNA expression for mTOR and 4E-BP1 in TCGA cohort.

After a median follow-up of 74.5 months (IQR 65.0-84.0), 27 patients (23.47%) developed BCR.  Table 3 shows the uni- and multivariate Cox regression analysis for predictive factors of BCR on the basis of immunostaining results. We demonstrated that weak staining of mTOR was associated with BCR (HR: 2.0; *p* < 0.05) after correcting for PSA and T stage. However, segregating into localization A and B, we found only in B a significant impact of mTOR on BCR (*p* = 0.0034 log rank).  Figure 5 shows the Kaplan-Meier curve for mTOR expression in localization B dichotomized by the median.

In the meta-analysis, 4 studies [14, 16–18] with 736 patients with PCa were identified as eligible for the forest plot after the evaluation of the association between immunostaining and BCR.

**Figure 6:**
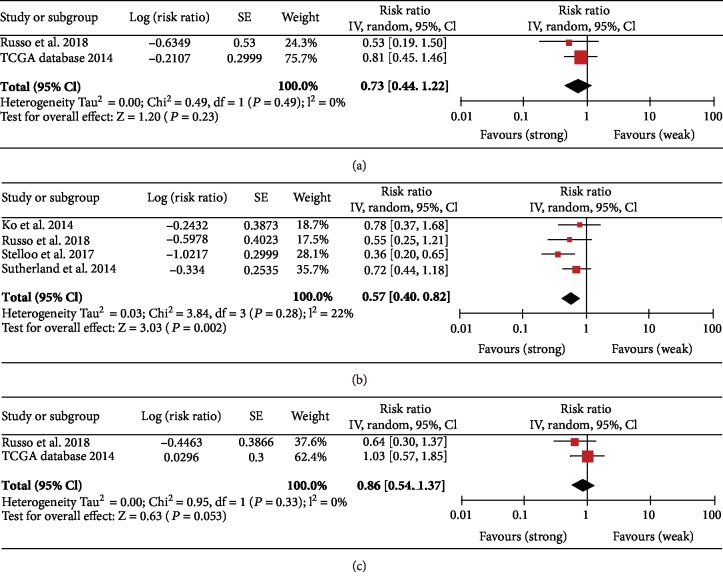
Association between staining results of mTOR (a), phosphorylated mTOR (b), and 4E-BP1 (c) and biochemical recurrence. CI: confidence interval; IV: inverse variance.

We found that the expressions of mTOR (risk ratio (RR) = 0.70; 95% CI 0.43-1.12; *p* = 0.13) and 4E-BP1 (risk ratio (RR) = 0.86; 95% CI 0.54-1.37; *p* = 0.53) were not statistically associated with BCR, while strong staining of p-mTOR was associated with a lower risk of BCR (risk ratio (RR) = 0.57; 95% CI 0.40-0.82; *p* = 0.002) (Figure 6).

## 4. Discussion

Prostate cancer is considered a heterogeneous disease, with some cases of rapid progression counterposed to those with a low potential of distant metastasis [19, 20]. Interestingly, the mammalian target of rapamycin (mTOR) plays a central role in regulating critical PCa cellular processes and tumorigenesis [21]. mTOR is a protein kinase that is present in two distinct complexes: mTOR complex 1 (mTORC1) and mTOR complex 2 (mTORC2) [21]. mTORC1 increases mRNA translation by the phosphorylation of eukaryotic initiation factor 4E- (eIF4E-) binding protein-1 (4E-BP1) [22], which is crucial for tumor growth [23, 24].

In the present study, we initially demonstrated that the expression of important regulators of the mTOR pathways differs in the tumor center, the tumor invasion front, the tumor-adjacent benign tissue, and the tumor-distant benign tissue. This underlines the importance of intratumoral heterogeneity with regard to the use of predictive and prognostic biomarkers. Interestingly, in the present study, the expression of biomarkers in the tumor invasion front showed a stronger association with outcome than the expression in the tumor center.

The results of our study indicate that disorders in the mTOR system may occur as early events in PCa later replaced by its decrease after tumor progression. This may be supported by the finding that low p-mTOR expression is associated with worse oncological outcomes in terms of BCR.

Performing in silico analysis of the PCa cohort of the TCGA, we found that 4E-BP1 protein levels were decreased in locally advanced disease and that the mRNA expressions of both mTOR and 4E-BP1 were decreased with high Gleason scores.

The findings of our study are supported by several other studies. Sutherland et al. showed that p-mTOR upregulation occurs early in the development of PCa and that the expression of p-mTOR is increased in putative precursor lesions of PCa [16]. Similarly, Evren et al. reported a decrease in p-mTOR expression from high-grade PIN through Gleason 7 to high-grade tumors in 179 prostate cancers [25].

Stelloo et al. showed that PCa patients with a high risk of relapse have low-mTOR-expressing tumors with an inactive mTOR pathway [17], and similarly, Muller et al. demonstrated that the loss of p-mTOR staining was significantly linked to early biochemical recurrence across various types of cancers [26].

The prognostic relevance of the mTOR pathway in PCa is widely unclear. For this purpose, we performed a meta-analysis of current literature data with a total of 897 patients, including our own cohort. Interestingly, we found a statistically significant association between strong p-mTOR staining and a lower risk of BCR, emphasizing the hypothesis of the overexpression of mTOR signaling in less aggressive PCa.

Considering these tissue-based marker results, we speculate that the mTOR pathway is involved in the early development of PCa and is downregulated in the progression of PCa. We also postulate a potential role of the p-mTOR tissue marker as a prognostic factor for BCR.

In fact, growing evidence shows that the mTOR signaling pathway plays an important role in the development androgen deprivation therapy resistance and stimulates tumor growth in the setting of castrated levels of testosterone [27]. In this context, the ATLAS group performed a whole exome sequencing molecular analysis of more than 300 primary PCa samples, allowing to introduce a molecular taxonomy in PCa tumors. The authors showed that about a quarter of localized prostate tumors displayed activating mutations of the PI3K/Akt/mTOR and MAPK signaling pathways [14, 28]. However, the use of mTOR inhibitors in patients with PCa has not shown significant success yet [29, 30].

One potential explanation for the lack of benefit is that PCa patients with “weak” expression of mTOR pathways showing adverse pathological features and a greater risk of BCR are unlikely to benefit from mTOR inhibitor therapies.

The present study has some important limitations. First, the sample size is limited, and larger studies are required in the future. Second, the control group was obtained from histologically normal-looking areas but not from tumor-bearing prostates. Moreover, we were not able to assess heterogeneity of several prostate cancer foci and also the relationship between protein expression and different Gleason score findings inside the tumor.

Furthermore, we did not stratify patients according to the presence of an aggressive Gleason score (≥8) or the presence of seminal vesicle invasion that may harbor significant worse outcomes during the follow-up. Finally, the Gleason score was not evaluated according to the last ISUP consensus.

However, to the best of our knowledge, this is the first study to address the effects of intratumoral heterogeneity on the expression of mTOR, p-mTOR, and 4E-BP1 and their prognostic relevance. The findings of our study should encourage the inclusion of the location of a sample within the tumor (e.g., relative to the tumor invasion front) as a potential factor that impacts the significance of a prognostic biomarker.

## 5. Conclusions

Intratumoral heterogeneity has an important impact on the expression of proteins of the mTOR pathway in PCa. A weak expression of p-mTOR is associated with adverse pathological features and worse oncologic outcome. Protein expressions at the tumor edge provide more prognostic significance than those at the tumor center.

These findings should be confirmed in other studies with the aim of tailoring treatment regimens.

## Figures and Tables

**Table 1:** Baseline characteristics of the patients.

Number of patients	*n* = 115
Age, median (IQR)	65.0 (60.0-69.0)
PSA, median (IQR)	9.0 (5.3-12.9)
Clinical lymph node metastasis, *n* (%)	5 (4.3)
Bilateral disease, *n* (%)	92 (80.0)
Pathological T stage, *n* (%)	
T2	78 (67.8)
T3a	17 (14.8)
T3b	20 (17.4)
Pathological Gleason score, *n* (%)	
6	62 (53.9)
7	45 (39.1)
8-10	8 (7.0)
Perineural invasion, *n* (%)	33 (28.7)
Positive margins, *n* (%)	25 (21.7)

IQR = interquartile range; PSA = prostate-specific antigen.

**Table 2:** Expression patterns of mTOR, phosphorylated mTOR, and 4E-BP1 (A) the tissue from the tumor center, (B) tumor tissue of the tumor invasion front tumor, (C) benign tissue adjacent to the tumor, and (D) benign tissue distant from the tumor.

Molecular markers	A	B	C	D	*p* value between all groups^∗^
mTOR, mean (SD)	9.1 (2.9)	8.8 (3.3)	5.0 (3.8)	6.8 (3.7)	<0.01
Phosphorylated mTOR, mean (SD)	7.4 (3.5)	7.2 (3.5)	2.0 (2.6)	4.6 (3.5)	<0.01
4E-BP1, mean (SD)	8.0 (3.3)	7.6 (3.2)	2.6 (2.0)	3.1 (2.1)	<0.01

^∗^ANOVA test. mTOR = mammalian target of rapamycin; 4E-BP1 = 4 eukaryotic-binding protein 1.

**Table 3:** Uni- and multivariate Cox regression analyses for the association between immunostaining expressions of mTOR (model 1), p-mTOR (2), and 4E-BP1 (3) and biochemical recurrence.

	Univariate HR (95% CI)	*p* value	Model 1	*p* value	Model 2	*p* value	Model 3	*p* value
mTOR, weak vs. strong staining	1.9 (0.9-4.0)	0.11	2.0 (1.1-4.2)	0.04	—	—	—	—
p-mTOR, weak vs. strong staining	1.8 (0.8-3.8)	0.12	—	—	1.1 (0.5-2.6)	0.74	—	—
4E-BP1, weak vs. strong staining	1.6 (0.7-3.4)	0.25	—	—	—	—	1.7 (0.8-3.8)	0.26
PSA (ng/ml)	1.1 (1.0-1.2)	0.01	1.1 (1.0-1.2)	0.04	1.0 (0.9-1.1)	0.09	1.1 (1.0-1.2	0.07
Gleason score, Gleason score ≥ 7 vs. <7	3.2 (1.1-9.2)	0.03	—	—	—	—	—	—
Pathological stage, pT3 vs. pT2	4.2 (1.9-9.0)	<0.01	3.8 (1.7-8.3)	<0.01	3.4 (1.5-7.9)	<0.01	3.8 (1.7-8.3)	<0.01
Positive margins, yes vs. no	3.7 (1.6-8.5)	<0.01	—	—	—	—		

mTOR = mammalian target of rapamycin; p-mTOR = phosphorylated mammalian target of rapamycin; 4E-BP1 = 4 eukaryotic-binding protein 1; BCR = biochemical recurrence.

## Data Availability

The data type used to support the findings of this study is available from the corresponding author upon request.
